# Embryonic Lethal Phenotyping to Identify Candidate Genes Related with Birth Defects

**DOI:** 10.3390/ijms25168788

**Published:** 2024-08-13

**Authors:** Bing Yan, Baoming Gong, Xue Wang, Yufang Zheng, Lei Sun, Xiaohui Wu

**Affiliations:** State Key Laboratory of Genetic Engineering and National Center for International Research of Development and Disease, Institute of Developmental Biology and Molecular Medicine, Collaborative Innovation Center of Genetics and Development, School of Life Sciences, Fudan University, Shanghai 200441, China; 20110700085@fudan.edu.cn (B.Y.); 22110700022@m.fudan.edu.cn (B.G.); wangxue_@fudan.edu.cn (X.W.); zhengyf@fudan.edu.cn (Y.Z.)

**Keywords:** congenital birth defects, embryonic lethal gene, mutation, phenotypic analysis, PBmice

## Abstract

Congenital birth defects contribute significantly to preterm birth, stillbirth, perinatal death, infant mortality, and adult disability. As a first step to exploring the mechanisms underlying this major clinical challenge, we analyzed the embryonic phenotypes of lethal strains generated by random mutagenesis. In this study, we report the gross embryonic and perinatal phenotypes of 55 lethal strains randomly picked from a collection of mutants that carry piggyBac (PB) transposon inserts. Gene Ontology (GO) and Kyoto Encyclopedia of Genes and Genomes (KEGG) analyses suggested most of the analyzed mutations hit genes involved in heart and nervous development, or in Notch and Wnt signaling. Among them, 12 loci are known to be associated with human diseases. We confirmed 53 strains as embryonic or perinatal lethal, while others were subviable. Gross morphological phenotypes such as body size abnormality (29/55, 52.73%), growth or developmental delay (35/55, 63.64%), brain defects (9/55, 16.36%), vascular/heart development (31/55, 56.36%), and other structural defects (9/55, 16.36%) could be easily observed in the mutants, while three strains showed phenotypes similar to those of human patients. Furthermore, we detected body weight or body composition alterations in the heterozygotes of eight strains. One of them was the TGF-β signaling gene *Smad2*. The heterozygotes showed increased energy expenditure and a lower fat-to-body weight ratio compared to wild-type mice. This study provided new insights into mammalian embryonic development and will help understand the pathology of congenital birth defects in humans. In addition, it expanded our understanding of the etiology of obesity.

## 1. Introduction

Congenital birth defects are structural, functional, or mental abnormalities present at birth, among which congenital heart disease [1], neural tube defects, and Down syndrome are the most common ones [2,3]. Congenital birth defects contribute significantly to preterm birth, stillbirth, perinatal death, infant mortality, and adult disability [4,5,6]. It is estimated that 6% of infants worldwide are born with a congenital birth defect, leading to 240,000 neonatal deaths within 28 days after birth and 170,000 deaths in children aged 1–59 months each year [7]. More efforts are needed to reduce the mortality rate, promote primary prevention, and improve the health of children with congenital birth defects.

The causes of congenital birth defects are complex. Both genetic and environmental factors play significant roles [8]. Although approximately 50% of congenital birth defects cannot be attributed to a specific cause, genetic causes still make a significant contribution to infant malformations. Certain congenital malformations can be prevented through screening and timely intervention. Early genetic diagnosis allows the effective identification of congenital heart defects and cleft palate conditions that are amenable to treatment with medication and surgery [9]. Therefore, deciphering the genetic causes of birth defects is of great significance for preventing and controlling birth defects.

The mouse is a valuable genetic model organism for biomedical research. It shares a very similar genetic background with humans, with around 90% of both genomes being partitioned into regions of conserved synteny [10]. The placentation and early embryonic development of mice are also similar to those of humans, making them ideal for studying embryonic phenotypes at the molecular, cellular, organ, or even organismal levels. It has been claimed that approximately 30% of genetic mutations would cause embryonic or perinatal death in mice [11,12]. However, more studies are required to add specific loci to this long list.

The piggyBac (PB) transposon is a powerful tool with various applications, such as random mutagenesis, the generation of transgenic animals, forward genetic screens, and more [13,14,15]. Previous studies have employed PB transposon-mediated mutagenesis to discover novel cancer genes in chronic leukemia and solid tumors [16,17]. PB transposon shows high efficiency in both human and mouse cells, including mouse germ cells, making it a valuable tool for generating animal models [18]. We generated more than 5000 mutant strains (PBmice) by jumping PB into the mouse genome several years ago [19]. A pilot genetic screen thereafter identified 14 unknown loci with metabolic abnormalities [20]. Here, we reported the phenotypic analysis results of 55 embryonic lethal strains generated by PB mutagenesis. Each line was examined at four developmental time points with paraffin sectioning and hematoxylin/erythrosine (HE) staining to reveal potential morphological defects. Bioinformatic analyses were performed to explore the pathological mechanisms underlying embryonic developmental defects. In addition, body weight and body composition were measured for wean heterozygotes to explore the potential effects of embryonic lethal mutations on postnatal health.

## 2. Results

### 2.1. The Phenotyping Pipeline and Identification of Lethal Phenotypes in Mice

**Figure 1:**
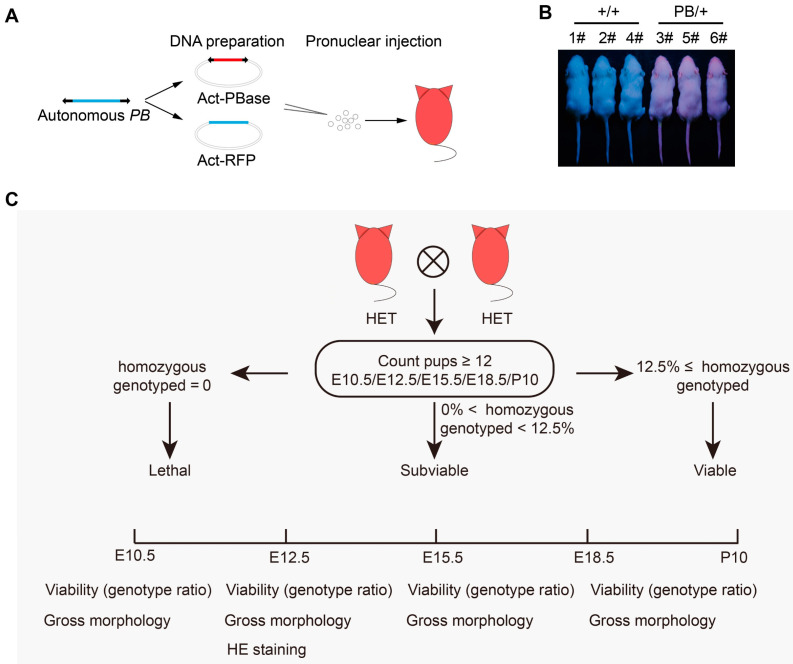
Generation of PB insertion mutations and embryonic lethal phenotyping pipelines. (**A**) Schematic of the PB mouse construct. (**B**) A representative image of *Gpr107*^PB/+^ mice and wild-type littermates at day P10 under a fluorescent lamp. (**C**) Embryonic lethal phenotyping pipelines were used to analyze the data.

To facilitate large-scale screens, the PB transposon utilized in PBmice was labeled with a red fluorescent protein (RFP) reporter (Figure 1A). Mice carrying this transposon exhibited red fluorescence when exposed to UV light (Figure 1B). During the mutagenesis process, 671 strains were recorded as lethal due to the fact that no homozygous mutants were recognized at the age of around 10 days after birth (P10) (Table S1). To further investigate the embryonic characteristics of lethal strains, we randomly selected 55 of these strains for a more comprehensive analysis of their embryonic features (Figure 1C). In general, breeding was set up between heterozygotes, and pregnant females were dissected at specific embryonic stages to obtain embryos for phenotyping. Yolk membranes were collected to genotype the embryos or pups. To determine the lethality stages, the proportion of WT/HET/HO offspring was calculated at specific time points. At the same time, we examined the gross morphology of these embryos. Furthermore, we performed histopathological analysis on E12.5 heterozygous or homozygous embryos of each strain.

**Figure 2:**
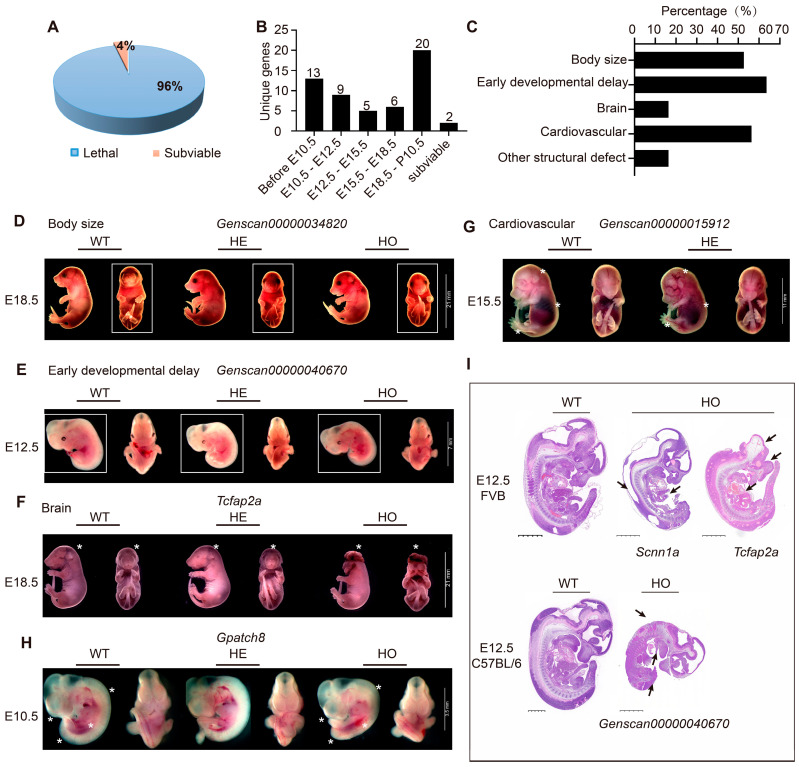
Identification of embryonic lethal phenotypes. (**A**) Proportions of subviable and lethal genes identified among 55 PB strains. (**B**) 55 PB strains were identified that exhibit lethality within a specific temporal stage. (**C**) The frequency of gross morphological phenotypes was assessed. (**D**–**H**). The gross morphology of the embryo exhibits typical structural defects, which are indicated by asterisks or boxes. (**I**). HE staining was performed on E12.5 living homozygous embryos of three genes (*Scnn1a*, *Tcfap2a*, *Genscan00000040670*) and wild-type. Black arrows indicate regions with typical structural defects, Scale bar: 1.25 mm.

Mouse embryogenesis is a complex process involving key events such as implantation, placentation, and organogenesis. Early lethality before E10.5 is often due to severe placental malformations. Abnormalities in cardiovascular development are common at E12.5 and E15.5, while respiratory and body wall abnormalities are frequently observed at E18.5 [12,21]. We examined embryos at E10.5, E12.5, E15.5, and E18.5, respectively. Among all 55 strains, 53 were confirmed to be embryonic or perinatally lethal, while the other 2 strains were subviable (Figure 2A, Table S2). Among 53 confirmed lethal strains, 13 (24.53%) died before E10.5, 9 (16.98%) died between E10.5 and E12.5, 11 (20.75%) died between E12.5 and E18.5, and 20 (37.74%) died between E18.5 and P10 (Figure 2B).

We observed the gross morphology of mutant embryos and recorded it with a structured set of Mammalian Phenotype (MP) terms, describing abnormalities in body size, growth/developmental delay, brain/vascular/heart malformation, and other structural defects (Figure 2C–G, Table S3). Developmental delay was the most frequently observed phenotype, accounting for 63.64% of 55 analyzed strains (Figure 2C,E). Abnormalities in cardiovascular development were the secondary common defect, frequently observed with craniofacial malformations (Figure 2F,G). Severe edema and local hemorrhage suggest circulatory disturbances. Short tails and curved spines, indicative of neural tube closure defects, were also observed (Figure 2H). Histopathologic analysis of E12.5 mutant embryos also revealed multiple typical defects, including abnormalities in the body wall, neural tube, head structure, cardiovascular defects, and tail development defects (Figure 2I).

### 2.2. Functional Enrichment Analysis of Embryonic Lethal Gene

**Figure 3:**
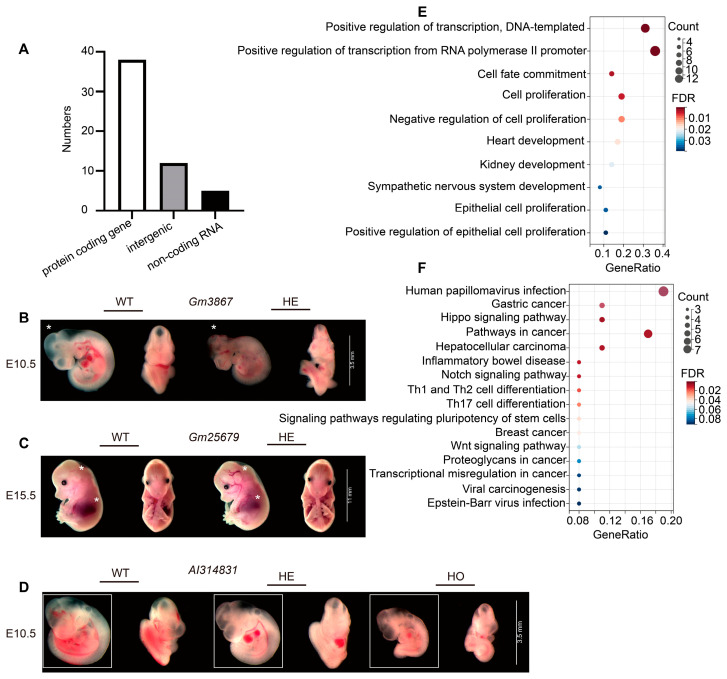
Enrichment analysis of mutated genes that caused embryonic lethality. (**A**) Sequence characterization of PB insertion site. (**B**–**D**) The gross morphology of the embryo. (**B**) *Gm3867*, (**C**) *Gm25679* and (**D**) *AI314831*. The asterisks or boxes indicate typical structural defects. (**E**) Top 10 enriched GO terms associated with embryonic lethality. (**F**) Enriched KEGG terms associated with embryonic lethality.

Among the PB mutants we analyzed, 38 hit protein-coding genes, 5 disrupted non-coding RNAs, and 12 were intergenic insertions (Figure 3A). Among the confirmed lethal strains, 23 loci, including *Gm3867*, *Gm25679,* and *AI314831*, were previously unknown to be associated with embryonic abnormalities (Table S4). Heterozygotes of *Gm3867* exhibited delays in central nervous system development at E10.5, *Gm25679* heterozygotes displayed cardiovascular abnormalities, and *AI314831* mutations caused significant developmental delay (Figure 3B–D).

To explore potential molecular mechanisms underlying observed embryonic lethality, we performed bioinformatic analysis for coding and non-coding genes among confirmed lethal loci. Enrichment Gene Ontology (GO) terms for biological processes, molecular functions, and cellular components were summarized in Figure 3E. Most embryonic lethality loci were enriched in biological processes such as DNA replication, RNA transcription, cell fate commitment, regulation of cell proliferation, and organ and system development. The pathways with the highest enrichment in Kyoto Encyclopedia of Genes and Genomes (KEGG) are shown in Figure 3F. KEGG category terms encompass a wide range of biological processes and pathways, such as gastric and breast cancer, Wnt, Notch, and Hippo signaling pathways, and EB and HPV infection response pathways. Wnt and Notch signaling pathways play vital roles in various cellular processes, such as cell proliferation, differentiation, and apoptosis [22,23]. In embryonic development, Wnt signaling is essential for establishing the embryonic axis, directing axonal projections in the central nervous system, organ development, and mesoderm [24,25]. Disruption of Wnt signaling can lead to failures in these crucial processes. Notch signaling is also important in the communication between the fetus and mother during implantation and placentation [26,27,28]. Abnormal Notch activity has been associated with impaired placentation. Our findings confirm the essential role of embryonic lethality genes, particularly those involved in Notch and Wnt signaling, in developing the heart and sympathetic nervous system.

### 2.3. Body Weight and Composition Analysis of Embryonic Lethal Gene Suggest Smad2 as a Metabolic Regulator

**Figure 4:**
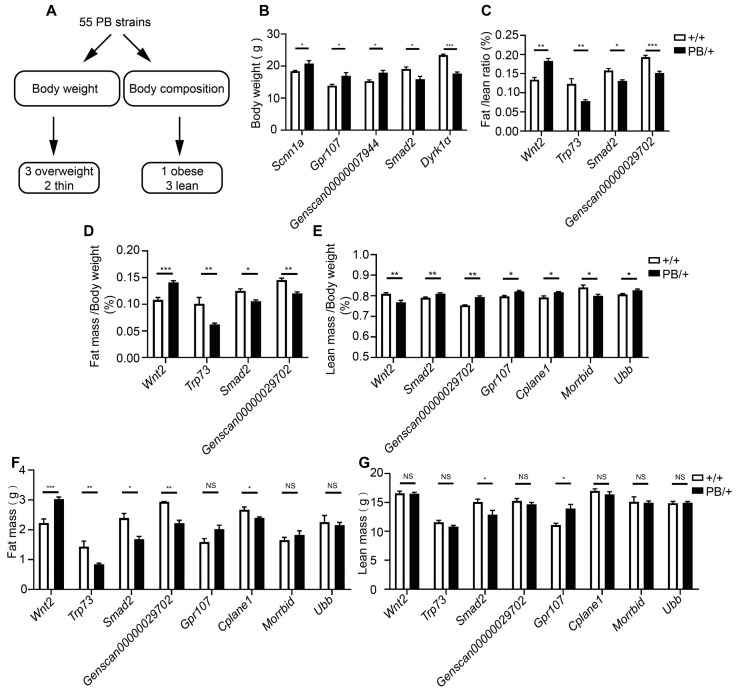
Body weight and body composition analysis. (**A**) Schematic diagram of experimental analysis. (**B**) Body weight of screened mutants. (**C**) Fat/lean ratio. (**D**) Fat mass to body mass ratio (**E**) and lean mass to body mass ratio are represented in the graph. (**F**) fat mass (**G**) and lean mass. The empty bars represent +/+ and the filled bars represent PB/+ (*n* ≥ 3). The data presented are the mean ± S.E.M. The statistical significance was determined to be * *p* < 0.05, ** *p* < 0.01, and *** *p* < 0.001 by Student’s t-test, NS, no significant difference.

Homozygous mutations in embryonic lethal genes cause embryonic lethality, whereas a 50% dose of normal genes is sufficient for embryonic development and survival. Nevertheless, haploinsufficiency of embryonic lethal genes can significantly contribute to the predisposition to metabolic syndrome and obesity in adulthood [29,30]. For instance, the homozygous knockout of *Ptcd1* is lethal during embryogenesis; however, heterozygous mice with diminished PTCD1 levels exhibit obesity during adulthood [30]. To explore more dominant loci involved in obesity. We examined body weight and body composition parameters in male heterozygotes of these 55 strains (Figure 4A). At the age of 4 weeks, mutants of *Scnn1a*, *Gpr107*, and *Genscan00000007944* were heavier than their wild-type littermates, while *Smad2* and *Dyrk1α* mutants became lighter (Figure 4B). More mutants exhibited abnormal body composition, although this did not eventually result in body weight change. We found that *Wnt2* mutants accumulated more fat (Figure 4C), consistent with the role of Wnt signaling as a key suppressive signal in adipocyte differentiation. Mutants of *Smad2*, *Trp73*, and *Genscan00000029702*, in another way, failed to build up regular levels of adipose tissue (Figure 4C,D,F). A lean mass abnormality was observed in mutants of *Cplane1*, *Ubb*, and *Morrbid* (Figure 4E,G). In conclusion, these findings suggest that genes necessary for embryonic development also play a role in the regulation of energy metabolism.

**Figure 5:**
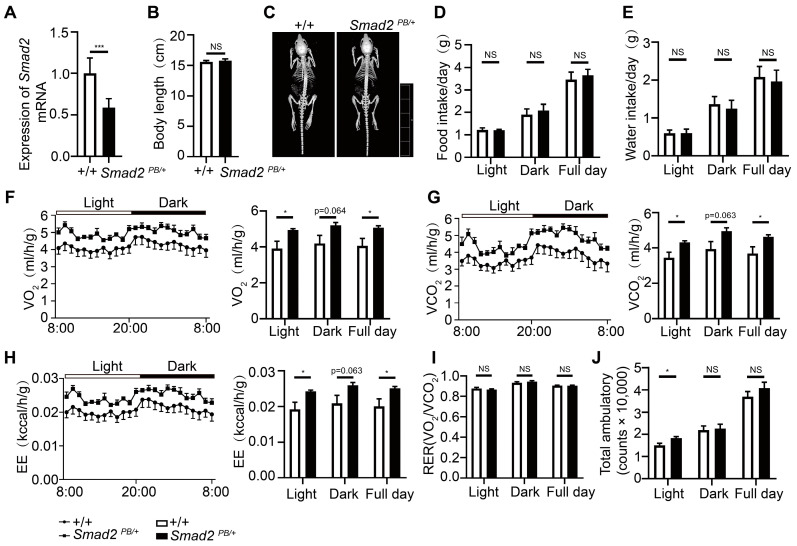
*Smad2* deficiency increases energy expenditure. (**A**) qRT-PCR analysis of *Smad2* in E10.5 embryos from wild-type (+/+) and *Smad2^PB/+^* mice. Empty bars (+/+) (*n* = 7) and filled bars (*Smad2^PB/+^*) (*n* = 8) were used to represent the data. Wild-type data were used as the statistical control. (**B**) The body length of +/+ and *Smad2^PB/+^* mice was measured. (**C**) CT images of +/+ and *Smad2^PB/+^* mice were taken. (**D**,**E**) At 4 weeks of age, food and water intake were assessed in both genotypes. (**F**) Metabolic cage studies were performed at 4 weeks of age to assess O_2_ consumption, (**G**) CO_2_ production, (**H**) energy expenditure, (**I**) RER, and (**J**) Locomotor activities. *n* = 5 mice per group. The data presented are the mean ± S.E.M. The statistical significance was determined to be * *p* < 0.05, and *** *p* < 0.001 by Student’s t-test, NS, no significant difference.

The role of *Smad2* has been reported in diseases such as congenital heart defects and growth restriction [31,32]. Nevertheless, it remains unclear whether *Smad2* is involved in metabolic regulation. We observed that a PB insertion in *Smad2* led to a 41.4% reduction in mRNA levels in E10.5 heterozygotes and embryonic lethality in homozygotes before E12.5 (Figure 5A). Compared with wild-type littermates, *Smad2^PB/+^* mice showed a 16.72% reduction in body weight and a 17.45% reduction in fat to lean ratio at the age of 4 weeks (Figure 4B,C). Meanwhile, their body length and skeleton morphology did not show significant differences (Figure 5B,C). The lean phenotype may result from an imbalance in energy metabolism, which could be caused by reduced energy intake or increased energy expenditure. To distinguish these possibilities, we first measured the food consumption of *Smad2^PB/+^* mice but failed to detect significant differences from that of their wild-type littermates (Figure 5D,E). We then assessed the metabolic parameters of *Smad2^PB/+^* mice with a metabolic cage system. We found *Smad2^PB/+^* mice had significantly higher oxygen consumption, carbon dioxide production, and energy expenditure compared with their wild-type littermates (Figure 5F–H). Meanwhile, the respiratory exchange rate (RER), an indicator of the substrate used for energy production, was not changed (Figure 5I). So was the locomotor activity of *Smad2^PB/+^* mice (Figure 5J). Taken together, these results suggest that higher energy expenditure led to a reduced fat-to-lean ratio and lower body weight in *Smad2^PB/+^* mice.

## 3. Discussion

Mouse genes showed a high degree of functional conservation with their human counterparts [33]. Lethal mutations in mice were often associated with human disease genes [33,34]. In this study, we analyzed the embryonic developmental phenotypes of 55 lethal strains at multiple time points. Among them, 12 (21.82%) have a human counterpart involved in disease processes (Table S5). Most of the analyzed mutations were confirmed to be embryonic or perinatal lethal, with two (*Wnt2* and *Pdzrn3*) being subviable. We detected short bodies in 52.73%, early developmental delays in 63.64%, brain defects in 16.36%, cardiovascular developmental defects in 56.36%, and structural abnormalities in other organs in 16.36% of the strains. Our study supports the idea that characterizing mouse embryonic lethal phenotypes not only provides valuable information for human birth defects but also offers new clues for the prenatal diagnosis of early developmental disorders in humans. Many of the mutations induced by PB insertion showed similar phenotypes as conventional gene-targeting alleles did, suggesting PBmice as a valuable resource for genetic screenings to answer disease and biological questions.

Protein-coding genes play a pivotal role in the complex process of mouse embryogenesis, encompassing crucial events such as implantation, placental development, and organogenesis [11]. Mutations in protein-coding genes have been identified as a significant contributing factor to embryonic lethality. Among the PB mutants, 38 mutations (69.09%) affected protein-coding genes. A total of 32 protein-coding genes associated with embryo lethality were identified, including *Tfam* (mitochondrial transcription factor A) [35,36] and *Smad2* [37].These mutations are believed to be significant in implantation, placentation, and cardiovascular development. Mutations in 12 loci, including *Tcfap2a* and *Pcsk5*, have been identified as contributing to various human diseases (Table S5). Notably, there are similarities in the mutant phenotypes observed in mouse models and humans. For example, mice carrying a mutation in the *Tfap2a* gene exhibit complete bilateral orofacial clefting. In humans, mutations in TFAP2A have been identified as a cause of branchio-oculo-facial syndrome (BOFS) and are linked to genes such as *IGF6*, *BCOR*, and *P63*, which are known to be connected with orofacial clefting [38]. Similarly, mutations in the *Pcsk5* gene result in the occurrence of VACTERL (vertebral, anorectal, cardiac, tracheoesophageal, renal, and limb malformations) in both humans and mice [39]. Furthermore, six loci were identified as essential genes for embryonic development but were previously unidentified (Table S4). For instance, the PB insertion in *Gm3867* resulted in homozygous lethality before E10.5, whereas heterozygotes exhibited a significant developmental delay in the central nervous system at E12.5 (Figure 3B). Thus, conditional knockout mouse models that deplete *Gm3867* levels temporally and specifically in specific tissues will facilitate the exploration of *Gm3867*’s role in development and disease pathogenesis. Further analysis of lethal PB mutants will yield new insights into gene function in mammalian embryonic development. Nevertheless, differences between mice and humans cannot be ignored, particularly with regard to the developmental rate and the development of the nervous system in mice. For example, there is a 1000-fold difference in the size of the cerebral cortex between mice and humans, as well as notable variations in the types, proportions, and distributions of cells [40]. It is essential to acknowledge the limitations of the current findings and to recognize the necessity of further research to gain a deeper understanding of human embryogenesis and to improve clinical translation.

The mammalian genome is also transcribed into non-coding RNAs (ncRNAs), while “non-coding” does not necessarily mean “non-essential” [41]. ncRNA is a variety of RNA species, including ribosomal RNA (rRNA), small nuclear RNA (snRNA), and others, which can be further divided into short ncRNA (<30 nts; miRNAs) and long ncRNA (lncRNA) [42,43]. ncRNAs are essential in diverse biological processes, such as RNA maturation, processing, gene expression, and protein synthesis [44,45]. Previous studies have shown that ncRNA plays a crucial role in both embryonic organogenesis and mature development. For example, overexpression of miR-1 during this process downregulates the HAND2 transcription factor, preventing expansion of the ventricular myocardium [46]. Among the PB mutants, five loci were identified as essential for the embryo’s development, comprising one snRNA and four lncRNA (Table S4). Prior research has indicated that snRNA functions with associated proteins to facilitate the splicing process. Abnormalities in this process can result in the development of familial Alzheimer’s disease (AD) [47]. We discovered that PB insertion in *Gm25679*, identified as a snRNA, resulted in homozygous lethality before E10.5 and that heterozygotes exhibited abnormalities in cardiovascular development at E15.5 (Figure 3C). Previous reports have indicated that embryos that die before E10.5 may be the consequence of severe placental malformations [21]. Placental insufficiency can result in fetal growth restriction or the emergence of specific abnormalities in the developing embryo. Our findings are consistent with this conclusion and point to a potentially crucial role for *Gm25679* in placental and organogenesis during embryonic development. These findings may have implications for the regulation of embryonic development and a comprehensive understanding of snRNA function. lncRNAs, another type of ncRNA, have been identified in several human diseases, such as myocardial infarction, liver cancer, and inflammatory bowel disease [48,49,50]. Reports on the role of lncRNAs in the regulation of embryonic development and survival are still limited. In the present study, we discovered that the mutation of *AI314831*, a conserved long non-coding RNA in humans and mice, could result in significant developmental delay and perinatal lethality (Figure 3D). These findings provide new examples of the contribution of lncRNA to embryonic development.

More than 69% of the mouse genome is composed of intergenic sequences [51]. They are believed to play crucial roles in various biological processes, such as gene expression regulation, chromosomal structure maintenance, and boundary element formation [52,53]. However, functional analysis of intergenic sequences was limited due to technical difficulties. We identified 12 intergenic mutations that could cause embryonic lethality in PBmice (Table S4). For instance, the insertion in *Genscan00000015912* caused homozygous lethality before E10.5 and obvious local hemorrhage (Figure 2G). Early lethality (E9.5–E14.5) is almost always associated with severe placental malformations, and placental defects strongly correlate with abnormal heart and vascular development [21]. Therefore, *Genscan00000015912* probably functions together in regulating cardiovascular and placental development in mice. The PB transposon system is widely recognized as a powerful tool for genetic engineering, due to its ease of mutation tracking and its ability to target the genome on a broad scale, as well as other beneficial features. Our findings are consistent with this viewpoint and reveal that more than 21.82% of PB insertions in PB mice are located in intergenic sequences. Therefore, it will be reasonable to analyze intergenic sequence function with PB mutants. However, we also need to conduct a comprehensive assessment of the PB transposon system’s effectiveness and consider the potential impact of random transgene insertion on endogenous genomic elements, particularly in genetic engineering applications involving individual mammals.

Obesity is a significant global public health concern [54]. It has been estimated that up to 70% of body weight variation is contributed by genetic variations [55]. Genome-wide association studies (GWAS) and meta-analyses have proposed dozens of BMI-related loci but only explain a small portion of BMI variation [55]. Traditional genetic analysis emphasized homozygous phenotyping. However, haploinsufficiency, a type of genetic dominance, is associated with a range of human inherited conditions due to the sensitivity of causal genes to dosage changes [56]. Previous studies have demonstrated that the haploinsufficiency of embryonic lethal genes exerts a significant influence on the predisposition to obesity in adulthood [30]. Among the PB mutants we analyzed, eight loci with significant variations in body weight or body composition were identified in heterozygous mutants (Figure 4B,C). Embryos homozygous for the *Smad2* mutation died before E12.5. Heterozygous *Smad2* mutant embryos exhibited a significantly lower ratio of fat mass and increased energy expenditure at four weeks of age. Prior research has demonstrated that SMAD2 proteins serve as pivotal regulators of TGF-β signaling, with a crucial role in the functions of both preadipocytes and mature adipocytes [57]. Therefore, we hypothesized that *Smad2^PB/+^* mice display a lean phenotype, possibly attributed to alterations in adipocyte development and maturation. Examining body weight or body composition in individuals with heterozygous mutations in embryonic lethal genes is critical for uncovering dominant loci that may contribute to obesity. These findings not only provided new hints for metabolic regulation but also suggested reconsidering the strategy in the genetic analysis of obesity in humans and model organisms.

In conclusion, our study provides new insights into embryonic development, organogenesis, and gene function in mammals. Meanwhile, our findings may also have implications for improving the clinical diagnosis of genetic diseases and reducing the incidence of congenital anomalies in humans.

## 4. Materials and Methods

### 4.1. Mice

All strains were generated using PB random insertion technology, as previously described [13,58]. In brief, the following series of steps are to be carried out in the specified order: DNA preparation, pronuclear injection, identification of positive founders, mapping of PB insertion sites, and establishment of single PB lines. The genomic sequence of PBmice can be found in Table S6. The PBmice collection contains over 5000 mutant mice, each carrying a single piggyBac insertion. Mutant strains were considered lethal if no homozygous mutants were present in at least 12 pups at P10, and subviable if their percentage was equal to or less than 12.5% of total offspring from heterozygous crosses. Wild-type and mutant mice were maintained in a specific pathogen-free facility with 12/12 h light/dark cycles on normal chow and water. A total of 55 strains of age-matched heterozygous mice were grouped in cages in a 1:1 (1:2) male/female ratio by batch between 6 pm and 8 pm, and the vaginal plug was checked at 8 am the next morning, counting the day of the vaginal plug as E0.5. Embryos and yolk membranes were collected for phenotyping and genotyping, respectively, at embryonic day 10.5 (E10.5), E12.5, E15.5, and E18.5.

### 4.2. Gene List Analysis

Gene lists were analyzed using the Ensembl genome browser tool (https://grch37.ensembl.org/Multi/Tools/Blast; acceded on 4 July 2023) and Mouse Genome Informatics (MGI) database (http://www.informatics.jax.org/; acceded on 4 July 2023). To distinguish between novel and previously reported knockout lines, alleles were screened to encompass ‘targeted’ and ‘null’ mutations exclusively, as these align with the alleles selected for this study. Additionally, a refinement process was conducted to incorporate only lines with documented phenotypic data indicating normal or abnormal characteristics.

### 4.3. Measurement of Body Composition and Body Length

At the age of 4 weeks, male mice were weighed and placed in a miniSpec NMR instrument (Bruker, Billerica, MA, USA;) to measure the amount and ratio of body fat, lean, and fluid composition. Body length was measured as the distance from the tail to the nose.

### 4.4. Metabolic Rate and Physical Activity

Oxygen consumption and physical activity were evaluated in 4-week-old male mice using CLAMS (Columbus Instruments, Columbus, OH, USA) according to the manufacturer’s instructions. The animals were acclimatized to the system for 20–24 h. Subsequently, parameters such as food and water intake, oxygen consumption (VO_2_), carbon dioxide production (VCO_2_), and total and ambulatory locomotor activity were recorded over the next 24 h. Energy expenditure (EE) and respiratory exchange ratio (RER) were calculated as previously described [20].

### 4.5. Morphological Observation of Embryos

Mouse embryos were performed as previously described [12]. Briefly, individual mouse embryos were placed in cell culture dishes and washed several times with pre-cooled 1 × PBS until the embryo surface was clear. Embryos from the WT/HE/HO groups were sequentially photographed under a stereoscopic microscope to obtain bright-field photographs of the sides of the embryos.

### 4.6. Histological Analysis

E12.5 HE/HO genotype mouse embryos were fixed in 4% paraformaldehyde at 4 °C overnight. They were then embedded in paraffin and sectioned through a gradient of ethanol dehydration, followed by sectioning at 5 μm thickness. Multiple sections were made and then stained with hematoxylin and eosin for general morphological observations.

### 4.7. Quantitative Real-Time PCR Analysis

According to the manufacturer’s instructions, total RNA was isolated from mouse embryos using the Trizol method (Invitrogen, Carlsbad, CA, USA). cDNA synthesis was performed using Prime Script™ RT reagent (Rr047a, TaKaRa Biotech, Shiga, Japan). The relative expression levels of target genes were determined using the AceQqPCR SYBR Green Master Mix (Q111, Vazyme Biotech, Nanjing, China). PCR reactions were performed in duplicate for each sample and analyzed on the LightCycler 480 Real-Time PCR System (Roche Diagnostics GmbH, Mannheim, Germany) with the following primers: *Smad2*: forward, 5′-ATGTCGTCCATCTTGCCATTC-3′, reverse, 5′-AACCGTCCTGTTTTCTTTAGCTT-3′; *Gapdh*: forward, 5′-AAATGGTGAAGGTCGGTGTG-3′, reverse, 5′-TGAAGGGGTCGTTGATGG-3′. Relative expression was calculated from the cycle threshold (Ct) using the following equation: relative expression = 2^−ΔΔCt^. *Gapdh* was used as an internal reference.

### 4.8. Bioinformatic Analysis

A KEGG pathway enrichment analysis of candidates was performed to discover pathways that were regulated by those embryonic lethal-related genes using the “GeneAnswers” package (http://www.bioconductor.org/packages/release/bioc/html/GeneAnswers.html; accessed on 8 November 2023). A GO enrichment analysis was performed using the “GOstats” package (http://www.bioconductor.org/packages/release/bioc/html/GOstats.html; accessed on 8 November 2023) to evaluate the functional and biologi cal significance of embryonic lethal-related genes in current research. Data visualization implemented in R language (version 4.2.3).

### 4.9. Statistical Analysis

Data were presented as mean ± SEM. Comparisons between the two groups were analyzed by Student’s *t*-test for independent samples. *p*-value ≤ 0.05 was determined to be statistically significant. Asterisks denote statistical significance levels, *: *p* < 0.05; **: *p* < 0.01; ***: *p* < 0.001.

## Data Availability

Data are presented in the article and Supplementary Materials.

## Supplementary Materials

The following supporting information can be downloaded at: https://www.mdpi.com/article/10.3390/ijms25168788/s1.
