# Correction: Yan et al. Embryonic Lethal Phenotyping to Identify Candidate Genes Related with Birth Defects. *Int. J. Mol. Sci.* 2024, *25*, 8788

**DOI:** 10.3390/ijms252413476

**Published:** 2024-12-16

**Authors:** Bing Yan, Baoming Gong, Xue Wang, Yufang Zheng, Lei Sun, Xiaohui Wu

**Affiliations:** State Key Laboratory of Genetic Engineering and National Center for International Research of Development and Disease, Institute of Developmental Biology and Molecular Medicine, Collaborative Innovation Center of Genetics and Development, School of Life Sciences, Fudan University, Shanghai 200441, China; 20110700085@fudan.edu.cn (B.Y.); 22110700022@m.fudan.edu.cn (B.G.); wangxue_@fudan.edu.cn (X.W.); zhengyf@fudan.edu.cn (Y.Z.)

In the original publication [1], Xue Wang was not included as an author. Bing Yan and Baoming Gong made equal contributions to the work, and are listed as the co-first authors. The corrected Author Contributions statement appears here. The authors state that the scientific conclusions are unaffected. The original publication has also been updated.
